# Kinetics of Carboxylic Acids Formation During Polypropylene Thermooxidation in Water Saturated with Pressurized Oxygen

**DOI:** 10.3390/polym17192696

**Published:** 2025-10-07

**Authors:** Vadim V. Zefirov, Polina S. Kazaryan, Andrey I. Stakhanov, Svetlana V. Stakhanova, Mikhail M. Ilyin, Ivan A. Godovikov, Elizaveta V. Shmakova, Andrey G. Terentyev, Alexander V. Dudkin, Elena P. Kharitonova, Marat O. Gallyamov, Alexei R. Khokhlov

**Affiliations:** 1 A.N. Nesmeyanov Institute of Organoelement Compounds, Russian Academy of Sciences, Vavilova St. 28, 119991 Moscow, Russia; kazaryan@polly.phys.msu.ru (P.S.K.); stakh@ineos.ac.ru (A.I.S.); mil@ineos.ac.ru (M.M.I.); pr0vider@ineos.ac.ru (I.A.G.); glm@spm.phys.msu.ru (M.O.G.); khokhlov@polly.phys.msu.ru (A.R.K.); 2 Department of Analytical Chemistry, Dmitry Mendeleev University of Chemical Technology of Russia, Miusskaya Sq. 9, 125047 Moscow, Russia; stakhanova.s.v@muctr.ru (S.V.S.); shmakova.e.v@muctr.ru (E.V.S.); terentev.a.g@muctr.ru (A.G.T.); dudkin.a.v@muctr.ru (A.V.D.); 3 Faculty of Physics, M.V. Lomonosov Moscow State University, Leninskie Gory 1-2, 119991 Moscow, Russia; harit@polly.phys.msu.ru

**Keywords:** plastic waste, chemical processing, polypropylene, thermal oxidative decomposition, carboxylic acids, wet oxidation

## Abstract

In this paper we study in detail the products formed during the process of water-assisted thermal oxidative decomposition (TOD) of polypropylene in the presence of pressurized oxygen. A set of techniques has shown that the main decomposition product in such a process is acetic acid with small amounts of other carboxylic acids (formic, propionic, succinic). The kinetics of carboxylic acid formation is studied by means of gas chromatography–mass-spectrometry as well as capillary electrophoresis, and the possible mechanisms behind the products formation are discussed. The role of water is considered based on the results obtained from substituting H_2_O with D_2_O in TOD. Compositions of residual oligomeric fractions as well as gas products are analyzed.

## 1. Introduction

Today, the world faces the problem of recycling a wide variety of waste [1,2]. More than 40% of produced waste belongs to various packaging materials, of which about 50% are polyolefins, namely polyethylene (PE) and polypropylene (PP) [3]. It is not surprising that methods for recycling these polymers are attracting close attention from the scientific community. It is known that plastic waste can be recycled through various ways: mechanical recycling, chemical processing and conversion of plastic into fuel [4]. Although mechanical recycling has already been brought to large industrial projects, it is currently limited by factors such as cost, necessity of presorting, deterioration in mechanical properties during recycling and unstable quality of recycled products [1,5]. In turn, the chemical processing of plastic waste is characterized by the greatest variability of specific processing methods [6,7,8]. One of the methods is pyrolysis, which is a thermochemical decomposition process occurring in an inert atmosphere in the typical temperature range of 350–800 °C [9]. Currently, this technology is the most common method of chemically processing of plastic waste [10]. This method is versatile and capable of processing a wide range of polymer materials while in the process, depending on the conditions various sets of products are formed [11,12]. Catalytic pyrolysis techniques allow the recycling of waste streams containing diverse plastics and produce high-value products such as carbon materials, fuel, and hydrogen [13,14,15]. However, most of the products obtained are of low quality (or low value added), contaminated [1] and usually destined for use as low-grade fuel, which leads to the wasteful loss of resources [16]. Eventually, the initial capital costs associated with the construction and operation of pyrolysis plants can be significant, which limits the benefits of this technology [17].

Another method for the chemical processing of plastic waste is thermal hydrolysis, which involves water as a reaction medium in the temperature range of 290–450 °C, resulting in the formation of gaseous, liquid and solid products [18]. The thermal process can be classified as hydrothermal liquefaction if water serves as the reaction medium or solvolysis if an organic solvent is used. As water approaches its critical point, significant changes in its properties occur, affecting the degradation rate, equilibrium and primary reaction pathways for decomposing plastics [19]. In its supercritical state (i.e., temperatures exceeding 375 °C and pressures exceeding 230 bar for water), water begins to exhibit non-polar solvent properties, which allows it to dissolve some plastics while providing an environment that is highly conducive for heat-transfer and diffusion. At the same time, water acts as a hydrogen donor, facilitating cracking reactions of polymers [20]. Hydrothermal treatment is a promising method for chemical processing of plastic waste, sometimes allowing the recovery of monomers (e.g., terephthalic acid, caprolactam) with properties similar to their original feedstock for use in polymer synthesis [21]. This approach is particularly suitable for condensation polymers because they typically contain hydrolyzable functional groups such as ester, ether, and amide ones [22]. Polyolefins, including PP and PE, present significant challenges for processing by subcritical hydrothermal liquefaction due to the lack of functional groups capable of undergoing chemical reactions under such conditions [23,24]. The lack of functional groups in polyolefins also results in lower reactivity compared to heteroatom-containing synthetic polymers such as polyethylene terephthalate, polycarbonates, and polyurethane, which have more reactive sites for hydrolysis and can thus be more easily depolymerized by subcritical hydrothermal liquefaction [25]. Therefore, supercritical conditions are preferred to initiate the decomposition of polyolefins. Processes under these conditions are classified as supercritical liquefaction or supercritical gasification. However, even under such “harsh” conditions, the main (more than 80%) decomposition product of polyolefins is a mixture of liquid hydrocarbons and paraffins, which makes such a process economically impractical [26,27].

Finally, another method of chemically processing plastic waste is the oxidative decomposition (OD) of polymers. The use of high temperatures (thermal oxidative decomposition (TOD)) or intense UV light (254 nm) may improve the polymer degradation rate by increasing the concentration of reactive oxygen species and initial polymeric radicals on the backbone [28]. During the oxidation of polyolefins, the introduction of hydroxyl and, carbonyl groups onto polymer chains occurs, and the functionalization degree as well as reactions pathways are strongly related to the type of the oxidant, pressure and temperature conditions, concentrations of the reagents, medium, etc.; there are some works in the literature describing the effect of oxygen (oxidizer) on the thermal decomposition of polyolefins, demonstrating, among other things, a decrease in the temperature of the onset of the destruction process in the presence of the oxidizer [29,30]. There are also works demonstrating the dependence of the distribution of the products obtained during OD on the oxygen pressure [31]. However, in these works this method is not considered as a promising one for plastic waste processing, since thermal oxidation of polyolefins, depending on the conditions, usually produces mixtures of products containing a large number of substances, including toxic ones. Thus, in one of the studies, the decomposition of polypropylene in an oxygen stream at temperatures of 120–280 °C was investigated. Indeed, it was shown that in addition to water and carbon oxides, the reaction products contained 47 different substances, including acetaldehyde, formaldehyde, acetone, crotonaldehyde, and acetic acid [32]. In another study, the same main products were obtained during the decomposition of polypropylene at 140 °C in a pure oxygen atmosphere [33]. At the same time, OD processes are promising for obtaining new functional polymers, value-added chemicals, and carbon-based materials [34,35,36,37,38]. There are approaches to polyolefin oxidation into carboxylic acids and short-chain oxidized PE [38,39,40,41]. In addition to the possible toxicity of the products, low selectivity of low-molecular-weight products, unexplored oxidation mechanisms, process scalability and practical implementations of the laboratory methods to real-life waste still remain as the challenges to be addressed [28,36].

Recently, our research group developed a new method for recycling polyolefin waste, which comprises both the concept of hydrothermal liquefaction and thermal oxidation. We showed that the combination of an oxidizing environment and pressurized water allows the conversion of polypropylene into a narrow range of acids dissolved in water, namely acetic acid (the main product), formic acid, and propionic acid [42]. The key benefit of the implemented method is the narrow range of useful products obtained, i.e., high selectivity. We have demonstrated the fundamental feasibility of this method but have not yet studied its mechanisms and features in detail. Having analyzed the results obtained in the work, we identified the reaction medium “water + oxygen under pressure” as the most promising. The aim of the work presented in this article is a deeper understanding and further development of the proposed method of water-assisted thermal oxidative destruction of polypropylene.

## 2. Materials and Methods

### 2.1. Raw Materials

Polypropylene (PP H030 GP/3) was provided by the SIBUR company (Moscow, Russia) in the form of round granules, (isotactic homopolymer, melt flow index 3.0 g/10 min). Pure oxygen (99.7%) was provided by Germes-Gas Company (Moscow, Russia). Milli-Q water (purity grade) was obtained using a Milli-Q Integral System (Millipore S. A. S., Molsheim, France). For all high-pressure experiments, a home-made stainless steel high-pressure autoclave with a titanium jacket and a PTFE seal was used. Additionally, a hollow PTFE cylinder with a lid was inserted into the autoclave to prevent contact between the reaction medium and the metal walls of the autoclave and eliminate any possible catalytic effect.

### 2.2. Chemical Processing of Polypropylene

**Figure 1:**
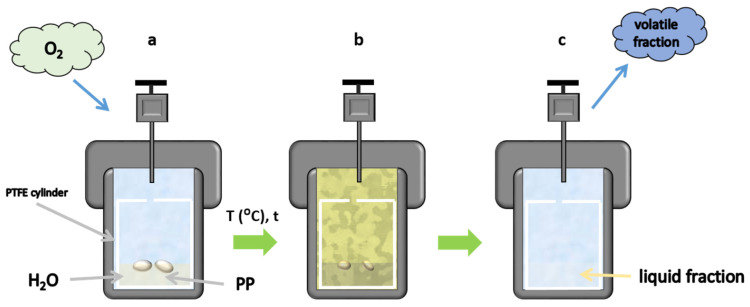
Schematic diagram of the experiment on the thermal oxidative destruction of polypropylene. (**a**) A PTFE cylinder with a lid, into which water has been poured in advance and a polymer sample has been added, is placed in the high-pressure autoclave. Oxygen is pumped into the autoclave, after which the autoclave is hermetically sealed. (**b**) The autoclave is heated to a certain temperature, which result in oxidative destruction of the polymer. (**c**) After a certain exposure time, the autoclave is cooled and decompressed, and eventually the remaining liquid (in some cases also residual solid) reaction products in the autoclave are to be extracted together with the PTFE cylinder.

The process of TOD of polypropylene (PP) was carried out according to the previously developed procedure. A 45 mg PP sample was placed in a 23 mL stainless steel high-pressure autoclave (Figure 1). In contrast to the method we previously described [42], a hollow PTFE cylinder with a lid having a 2 mm diameter hole in the center was additionally inserted into the high-pressure autoclave. This improvement was made to eliminate the influence of the autoclave walls, since our preliminary experiments showed that depending on the cleanliness of the autoclave walls, the resulting decomposition products can vary significantly. In addition, this experimental design allowed for accurate measurement of residual masses of oligomeric fractions after decomposition. After placing the polymer, 1 mL of Milli-Q water was added to the autoclave. The autoclave was hermetically sealed and pressurized with 20 bar oxygen. The polymer mass and oxygen pressure were also different from those used in our previous work [42]. New parameters were chosen to obtain the most reproducible homogeneous results, taking into account the placement of the PTFE cylinder in the autoclave (Figure S11). Additional studies were also conducted, which showed that a decrease in oxygen pressure leads to a slowdown in the process and an increase in the yield of the residual oligomeric fraction (Figure S10). An increase in oxygen pressure did not lead to significant changes in the process but only complicated its conditions. After filling, the autoclave was placed in a thermostat at 150 °C for a specified time (from 1 to 72 h). After exposure, the autoclave was removed from the thermostat, cooled to room temperature, decompressed, and then opened; the resulting products were collected for analysis. Similar experiments were conducted replacing water H_2_O with heavy water D_2_O.

### 2.3. Characterization of Liquid Products 

The products obtained during destruction were usually an aqueous solution of acids with the presence of an oligomeric fraction and a gas phase. The obtained liquid fraction was analyzed using a set of methods. To determine the total acidity of the samples, potentiometric acid-base titration with a 0.1 M NaOH standard aqueous solution as a titrant was carried out. An automatic titrator ATP-02 (Akvilon, Podolsk, Russia) with a combined glass pH electrode was used.

Nuclear magnetic resonance (NMR) and gas chromatography–mass-spectrometry (GC-MS) methods were used to analyze the polymer destruction products in the aqueous fraction. The analysis was carried out on a gas chromatograph with a Chromatec-Crystal (Chromatec, Yoshkar-Ola, Russia) mass-spectrometry detector. The experimental conditions are described in more detail in the Supplementary Materials.

The NMR spectra (^1^H and ^13^C) of polypropylene degradation products were obtained on a Bruker Avance II 600 spectrometer with operating frequencies of 600.22 and 150.93 MHz. Using water suppression by composite pulses, 1H spectra were recorded. 

Capillary electrophoresis was used to quantify the content of carboxylic acids. A capillary electrophoresis system CAPEL-105 M (Lumex, Moscow, Russia) equipped with a spectrophotometric detector and a quartz capillary tube (internal diameter 75 μm, effective length 50 cm, total length 60 cm) and Elforun^®^ 1.0 software (Lumex, Moscow, Russia) was used. The experimental conditions are described in more detail in the Supplementary Materials.

UV spectra of aqueous solutions of the liquid samples with 160-fold dilution were recorded in 190–900 nm range with a resolution of 2 nm at room temperature by means of an Unicam Helios alpha spectrometer (Thermo Fisher Scientific, Waltham, MA, USA), using a 10 mm path length quartz cuvette. A Milli-Q purified water probe was recorded as a baseline.

To confirm the presence of peroxide radicals and hydroperoxides in the liquid fractions obtained in the TOD process, analytical tests were carried out with aqueous solutions of potassium iodide (0.1 M) using starch as an indicator.

In addition, to confirm the presence of unsaturated compounds and aldehyde groups in the liquid PP decomposition products, analytical tests with an aqueous solution of bromine (0.18 M), potassium permanganate (0.025 M) and Cu(OH)_2_ in NaOH solution were carried out in accordance with Ref. [43].

### 2.4. Characterization of Solid Products 

To isolate residual solid polymer destruction products, the aqueous solution was dried at 35 °C for 5 days and then additionally evacuated at 40 °C for 3 days. Such a “soft” temperature regime made it possible to avoid the additional oxidation of the products, which was observed otherwise. The obtained products were characterized using elemental analysis, thermogravimetric analysis (TGA), differential scanning calorimetry (DSC), Fourier IR spectroscopy (FTIR), and gel permeation chromatography (GPC).

Elemental analyses of solid polymer destruction products were performed using a Vario MICRO Cube Elementar Analyzer (Elementar Analysensysteme GmbH, Langenselbold, Germany) and the content of carbon, hydrogen and nitrogen was determined.

TGA and DSC were performed using a NETZSCH STA 449 C device (Netzsch, Selb, Germany). All samples were placed in Al crucibles. The samples were heated from 30 to 590 °C at a rate of 5 °C min^−1^ in air.

FTIR data was collected using a Nexus spectrometer (Thermo Fisher Scientific, Waltham, MA, USA). The solid phase was analyzed after preparing compressed KBr tablets. Transmission spectra were recorded from 650 to 4000 cm^−1^. Each spectrum is an average of 64 scans with a resolution of 1 cm^−1^. For each sample type, three separate samples were tested to verify the reproducibility of the results.

GPC analysis was performed using a Knauer liquid chromatography system (Smartline series, Berlin, Germany) and a Phenomenex Phenogel column (300 × 7.8 mm, 5 μm, 1000 Ȧ). N-methylpyrrolidone was used as an eluent, with a flow rate of 1 mL/min. The column thermostat temperature was 25 °C, and a refractometer was used as the detector. The column was calibrated under these conditions using polystyrene standards. Data were received and processed using the Ecochrom software package (Techlab-M, Moscow, Russia).

Additionally, oligomeric residues of the destruction process were studied gravimetrically. For this purpose, a separate series of samples was prepared and evacuated at 90 °C. This allowed the achievement of more complete removal of water (the difference in mass of relatively “soft” evacuated samples was more than 2 times).

In order to confirm the presence of enol structures in the oligomeric residues, the evacuated samples were dissolved in 3 vol.% aqueous solution of anhydrous FeCl_3_. Its color was compared with the color of initial FeCl_3_ solution in H_2_O.

### 2.5. Gas Phase Characterization

To determine the mass of gaseous products formed during the process, the autoclave was weighed before and after decompression. To analyze the composition of the gas phase, the autoclave outlet was connected to a gas chromatograph Chromatec-Crystal 5000 (Chromatec, Yoshkar-Ola, Russia) through a capillary system, and decompression was carried out directly into the device.

## 3. Results and Discussion

### 3.1. Overview of the Products of the TOD Process in H_*2*_O/O_*2*_ and D_*2*_O/O_*2*_ Mixture

**Figure 2:**
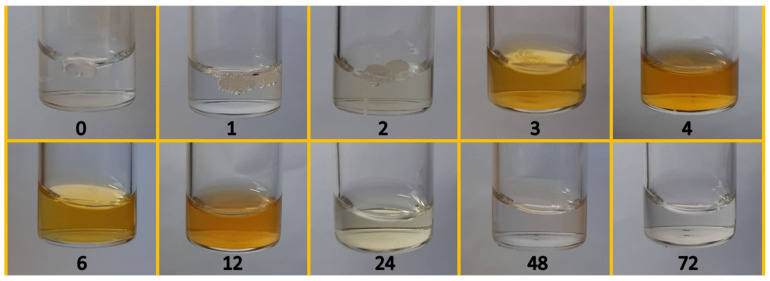
Photographs of samples obtained by TOD of PP in H_2_O/O_2_ mixture at different destruction times (in hours).

In contrast to the previously described methods of polypropylene destruction in the presence of dry oxygen as an oxidizer in the literature [32,33], the main products of our method are carboxylic acids. Indeed, carboxylic acids, especially acetic acid, are the main partial oxidation products of wet oxidation processes [44,45]. The expected mechanism of their formation will be discussed in detail below. In all experiments, the process parameters such as water, polypropylene and oxygen concentrations, pressure and temperature in the autoclave were fixed (see Section 2.2). By visual observation of the products at different degradation times, one can reveal the following stages of the thermal oxidation process in the H_2_O/O_2_ medium (Figure 2): the gradual oxidation of polymer to oligomer molecules with gradual formation of the carboxylic acids and volatile fractions (mostly CO_2_, see Section 3.6) leads to the visual disappearance of the polymer and the formation of a mixed solution of carboxylic acids and hydrophilic (water- (and ethanol-) soluble) oligomer residues, which are ultimately oxidized to carboxylic acids and volatile fractions. It is important to note that the polymer observed at 1 and 2 h of destruction is not colored but remains cloudy. At 3 h of destruction, visible polymer residues in the solution are minimal.

The solutions obtained were optically transparent, but with a yellow tint varying in intensity. During thermal oxidation, the mixture acquires an increasingly saturated hue; this may indicate an increase in the concentration of water-soluble products containing chromophores. It is important to note that even for 72 h of destruction under identical conditions, the samples could differ slightly in color intensity (Figures S1 and S2). Samples with the largest deviations in color and corresponding visible spectra were obtained after 12 h of TOD (Figure S2). These differences in the kinetics of formation of the intermediate products (which can be judged by different intensity of color) may be related to their sensitivity to the slightest changes in reaction conditions, which is typical for processes occurring via radical mechanisms. At the same time, the masses of dried residues (discussed below) at such times differ only slightly, as do the concentrations of acids in solutions (discussed below). Therefore, it can be assumed that the quantitative presence of substances imparting color to the samples is low. The UV spectra were measured for diluted aqueous solutions of the samples (Figure S2). Bands with absorption maxima at 200–220 nm UV most likely correspond to the electronic π→π* and n→π* transitions occurring in compounds containing conjugated double bonds and carbonyl groups [46]. 

When the liquid PP decomposition products interact with 0.18 M of bromine solution and 0.025 M of potassium permanganate solution, a rapid discoloration of the solutions is observed, which confirms the presence of double C=C bonds as well as hydroxyl and carbonyl functional groups in the structure of the products (Figure S8). 

In addition, the reaction of the liquid PP decomposition products with Cu(OH)_2_ in NaOH solution was carried out (Figure S7). For the liquid fractions obtained in the 6 h TOD process, a brick-red color due to Cu_2_O formation was observed, which indicates the unambiguous presence of aldehyde groups in the reaction mixture. At the same time, for the liquid fractions obtained in the 72 h TOD process, the reaction gives a negative result.

### 3.2. The Composition of the Liquid Products Obtained in the Water-Assisted TOD Process of PP

Despite the visual differences in the solutions obtained in the water-assisted TOD process, the analysis of the liquid fraction composition revealed identical substances in approximately the same proportions in all experiments performed under identical conditions. Using GC–MS, the following substances were identified in the composition of the products after 72 h of destruction: formic acid, acetic acid, propionic acid, succinic acid, methylsuccinic acid. The main products were acetic acid and formic acid; the remaining substances were identified in trace amounts. An overview of the GC–MS spectrum is given in the Supplementary Materials (Figure S4, Table S2).

**Figure 3:**
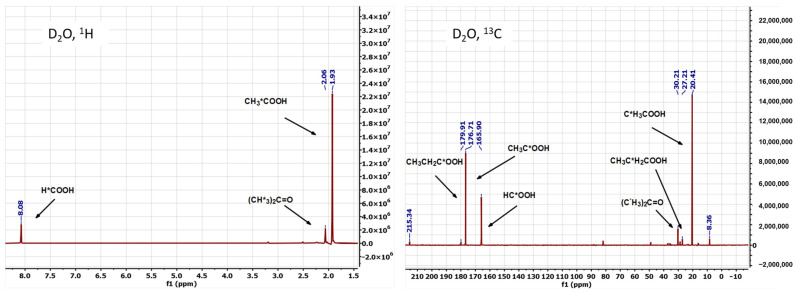
^1^H (left) and ^13^C (right) NMR spectra with suppressed water signal for sample obtained by TOD of PP in H_2_O/O_2_ mixture for 3 h.

For these types of samples, NMR spectra (^1^H and ^13^C) were also obtained (see Figure 3). The ^1^H spectra of the decomposition products contain signals at 1.93, 2.06, and 8.08 ppm corresponding to CH_3_- (acetic acid), CH_3_- (acetone), and H-C- (formic acid). The ^13^C spectra have signals in the region of 20.40 ppm, 28–32 (CH_3_- and -CH_2_- groups), 165, and 176–179 ppm (-COOH). In ^13^C spectra there are signals in the region of 8.36, 25–30, and 215 ppm, which are difficult to identify unambiguously. Comparison of ^1^H and ^13^C spectra allows the identification of the presence of compounds such as formic acetic, propionic acids, and acetone, which was not detectable by GC-MS. Overall, the results of both methods were similar.

### 3.3. Possible Mechanisms of Water-Assisted TOD of PP

**Scheme 1:**
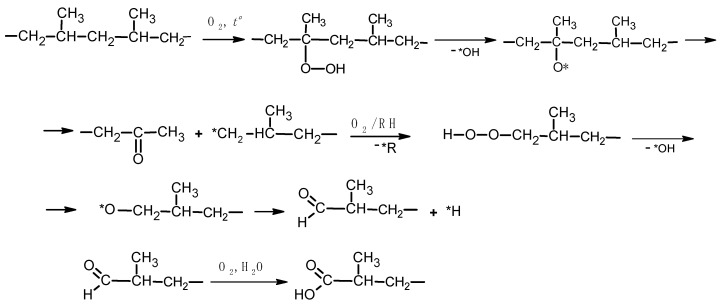
Possible radical reaction pathway leading to the forming of carboxylic acid at PP oxidation.

One may assume that, at the beginning of the process, a radical mechanism of destruction takes place during oxidation. This mechanism includes the following stages [33,47]. Oxygen firstly oxidizes the tertiary carbon of PP, consequently forming peroxide radicals (ROO^•^) and hydroperoxides (ROOH) after abstracting hydrogen from the chains. The β-scission mechanism of hydroperoxide decomposition leads to chain cleavage, producing shorter-chain carbonyl compounds (ketones and aldehydes) and alkyl radicals [47,48]. For example, in the oxidation processes of isotactic PP both in air at 225 °C and under 375 nm UV light at 35 °C, the decomposition of hydroperoxides via the β-scission mechanism (following the O-O bond scission) may lead to the formation of ketones (especially methyl (chain-end) ketones) as the principal carbonyl product [49]. Then the obtained shorter-chain carbonyl compounds can oxidize to carboxylic acids: for example, acetic acid can be produced from the attack of reactive species on methyl ketones [50]. The formation of carboxylic acid can be related to the oxidation of the methylene radical formed in the β-scission process via the formation of an aldehyde [51]. In a recently published work [52] devoted to the hydrothermal decomposition of PP, the authors, using DFT calculations, proposed that an alkyl radical forming from hydroperoxide in the β-scission process may be hydrated to form an alcohol and further oxidized to form a carboxylic acid. Following this consideration, possible reaction pathways are presented in Scheme 1. 

**Scheme 2:**
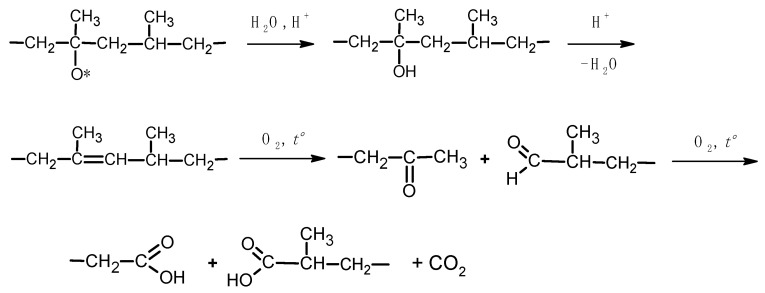
Possible ionic reaction pathway leading to the formation of carboxylic acid during PP oxidation.

When an acid, which can serve as a catalyst for the ionic reactions, appears in the reaction mixture, dehydration processes with the removal of hydroxyl groups start. The possibility of dehydration is supported by the ability of subcritical water to act as an acid catalyst [53,54]. Moreover, since the products of the TOD process contain CO_2_ (see Section 3.6), carbonic acid may form in the aqueous medium and additionally catalyze the dehydration [55]. This reaction pathway may explain the presence of a significant number of unsaturated compounds in the liquid fractions obtained in the TOD process (see qualitative reactions in Section 3.1). Ionic reactions in an acidic environment are accompanied by isomerization, migration of protons and methyl groups, and repeated hydration and dehydration processes, which lead to the formation of new products [56,57]. The presence of excess oxygen leads to the oxidation of unstable intermediate products and the formation of carboxylic acids at the end of the reaction (Scheme 2).

Thus, when conducting TOD in hydrothermal conditions, both radical and ionic processes may occur, leading predominantly to the formation of monocarboxylic acids. These acids can be formed by oxidation of aldehydes and ketones. In our system, the presence of compounds with aldehyde groups in liquid fraction was confirmed by standard qualitative reactions (Section 3.1).

The acetic acid is the most stable and dominant intermediate product in water-assisted processes of polypropylene thermooxidation. Indeed, in Ref. [58] the catalytic autoxidation of polypropylene in water as well as in acetic acid gave significant yields of 63% of acetic acid and acetone. It is known that aliphatic hydrocarbons autoxidize to aliphatic acids [59], which further break down to acetic acid [60,61]. Acetic acid is the most stable aliphatic acid towards autoxidation and is commonly used as an autoxidation solvent [61]. Acetic acid can be produced during the degradation of organic wastes under hydrolytic and oxidative conditions (400 °C, 276 bar) instead of complete oxidation to CO_2_ and water [62]. Acetic acid can also be oxidized under hydrothermal conditions, but the rate of this process is relatively low [63,64], since it is one of the most inert solvents in an autoxidation environment [61]. This stability can be attributed to the high activation barrier and low O_2_ reaction order for acetic acid oxidation even under supercritical water conditions (425–600 °C and 246 bar) [65]; further oxidation requires C–C scission from acetate, which is kinetically slow in hot compressed water [66].

### 3.4. The Kinetics of the Monocarboxylic Acids Formation in the Water-Assisted TOD Process of PP

**Figure 4:**
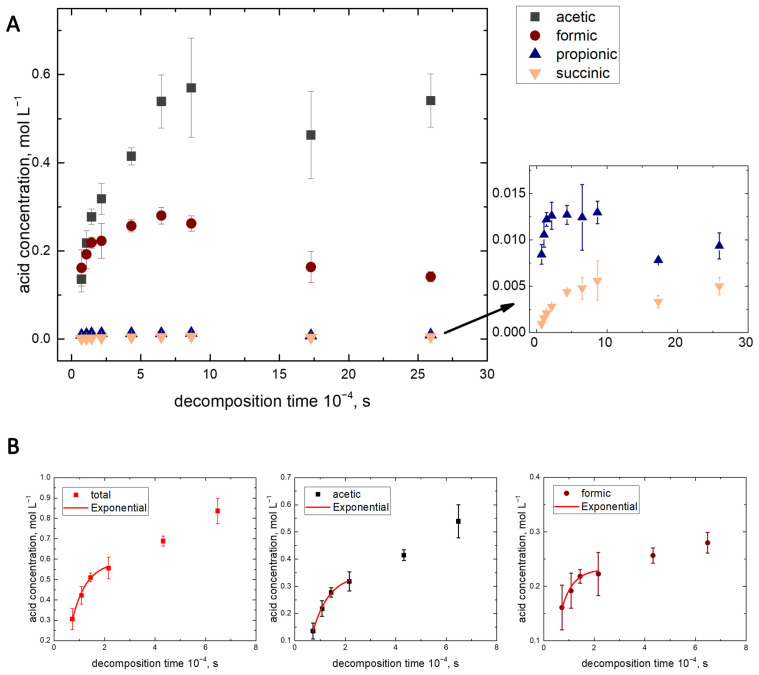
(**A**) Dependence of concentrations of the main destruction products of polypropylene: acetic, formic, propionic and succinic acids, depending on the destruction time. (**B**) The approximation of the acid formation kinetics with first-order (exponential) models at 2–6 h.

The identified acids were then quantitatively analyzed by capillary electrophoresis (Figure S5, Table S3). A series of experiments with different exposure times was conducted to determine the kinetics of polypropylene destruction reactions. Figure 4 shows data on the content of the main reaction products depending on the destruction time. It should be noted that the decomposition of polymers is a chemically complex process, in which several simultaneous reactions may occur, so a more accurate analysis of the kinetics of the products and intermediates may be required to postulate mechanisms. Moreover, in this paper, we focus on the TOD kinetics of one type of commercial isotactic polymer, so a more detailed study of the influence of molecular weight distribution, crystallinity degree, additives, etc., which may also affect the decomposition rate of PP, is beyond the scope of this work.

Let us consider the kinetics of the formation of acetic, formic, propionic, and succinic acids at the initial stage of 2–6 h. By 6 h, the solid polypropylene granules disappear, and an aqueous acidic solution of oligomers is obtained in the autoclave. As discussed above, the early stage of the TOD process may proceed via a radical mechanism. During the radical decomposition of polyolefins, the rate of formation of low-molecular products is proportional to the concentration of hydroperoxides (ROOH) [48,67], since low-molecular-weight products are mainly formed by the decomposition of hydroperoxides with the following formation of alkoxide radicals (which are the main source of low-molecular products) (see Scheme 1). One can consider the following basic mechanisms of the decomposition of hydroperoxide [48,67]:

(1) $$ROOH\overset{{k^{\prime }}_{d}}{\to }RO*+OH*$$

(2) $$ROOH+RH\overset{k_{d}}{\to }RO*+R*+H_{2}O$$

(3) $$2ROOH\overset{{k^{\prime \prime }}_{d}}{\to }RO*+H_{2}O+{{RO}_{2}}^{*}$$

where RH is monomer units of polymer, R*, RO*, RO_2_* are alkyl, alkoxide and peroxide macroradicals, correspondingly, and *k*_*d*_, *k*′_*d*_, *k*″_*d*_ are the constants of ROOH decomposition rate.

The most probable route of ROOH decomposition is the reaction of hydroperoxide with monomer units of the polymer (2) or with functional groups formed during oxidation [68]. The kinetics of hydroperoxide decomposition during the oxidation of polyolefins do not obey any simple kinetic law, but at the initial stage, the process is satisfactorily described by the first-order law [68]. Some authors presume that the prevailing first-order kinetics in condensed matter is the consequence of the heterogeneity of the polymeric matrix [69]. In Ref. [70] it was shown that the kinetic curves of the hydroperoxide decomposition of PP during oxidation at different oxygen pressures in the initial stages are slightly better described by a first-order reaction than by n = 1.5 or 2. The reaction of the formation of inactive products can be written as follows [68]:

(4) $$ROOH+RH\overset{k_{d}}{\to }\sigma R*+inactive\;products,$$

where σ is the radical yield.

It is important to note that the simplified reaction (4) does not take into account the entire complex mechanism of the transformation of OOH functional groups, and in this case, the concepts of “reaction order” and “rate constant” are only nominal. Following the idea of the first-order decomposition reaction of hydroperoxides, reaction (4) can also be considered a first-order reaction with respect to ROOH with an “effective” rate constant *k*_*eff*_ = *k*_*d*_[RH], where [RH] is a molar amount.

In Refs. [71,72] it was experimentally shown that the formation of carbonyl groups with the thermooxidation time of polyolefins had an exponential increase in the early stages of oxidation, which is followed by a linear increase in the later stages. Assuming that the first order of the reaction is also fulfilled for carboxylic acid formation (the main products in the TOD process under study containing carbonyl groups) at the early stages and that the [σR*] in reaction (4) is close to constant, we can obtain the following:

(5) $$-\frac{dROOH}{dt}=k_{eff}\sim \frac{d\left[carboxilyc\;acids\right]}{dt}=k_{acid}$$

The approximation results are given in Table 1. The obtained “effective” rate constants $k_{acid}$ of acid formation: $k_{total},\;k_{acetic},k_{formic},$ can be compared with the *k*_*eff*_ rate constants from the literature: for the PP with ROOH obtained via oxidation at 130 °C, the effective constants of hydroperoxides decomposition *k*_*eff*_ in vacuum can vary from 0.4 to 14 × 10^−4^ s^−1^ [68].

It is known that the reaction of molecular oxygen with polymers is an autocatalytic oxidation (autoxidation) reaction [48,67,68]. The autocatalytic process provides a good description of the processes of the degradation of polyolefins [73,74,75]. An autoxidation reaction starts slowly at first, possibly with a short induction period, followed by a gradual increase in its rate of oxidation (associated with the formation and build-up of hydroperoxides), and then subsiding; this dependence is well described by a sigmoidal curve (a logistic function) [76]. From this point of view, since hydroperoxide formation and decomposition are closely related to sigmoidal-like O_2_ absorption kinetics, the kinetics of carboxylic acid formation in the proposed TOD process can also be governed by autocatalytic equations. Carboxylic acid products may catalyze further decomposition of polyolefins [77], which leads to the corresponding formation of acids. 

After 6 to 18 h of TOD, the rates of acid formation seem to be constant and do not depend on acid concentration. For formic acid, a gradual decrease in concentration is observed after 20 h, which is most likely due to its further dehydration or oxidation:

HCOOH→CO + H_2_O; (6)

HCOOH + ½ O_2_ →CO_2_ + H_2_O. (7)

In addition, in Ref. [78] it was shown that the presence of acetic acid promotes decarboxylation and oxidation of the formic acid in the wet oxidation process. These processes increase the selectivity of acetic acid formation in the proposed polypropylene decomposition method. In general, the observed declines after 20 h in the acid concentration-time dependencies (Figure 4) can be associated with oxidation and decomposition of acids.

### 3.5. The Composition of the Solid Products Obtained in the Water-Assisted TOD Process of PP

**Figure 5:**
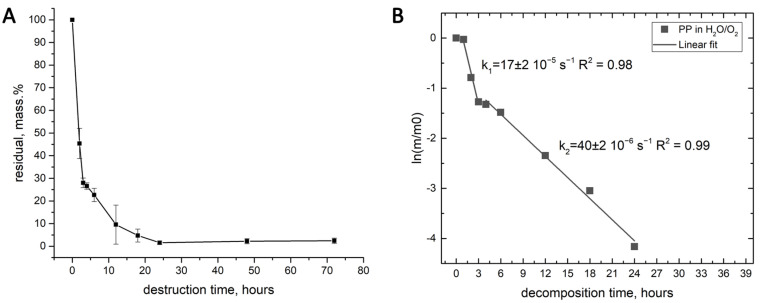
(**A**) Dependence of the mass of the residual solid fraction on the destruction time. The mass is given as a percentage of the initial mass of polypropylene granules. (**B**) The approximations of the relative time dependencies of the residual solid fraction by a first-order (linear) model for the TOD process in H_2_O/O_2_ mixture.

In addition to the liquid reaction products, we also studied the residual solid fraction. After a destruction time of 2 h, the samples still contained clearly visible polypropylene granule residues (Figure 5A). At the same time, the aqueous solutions acquired a yellow tint. After 3 h of destruction, there were practically no visually noticeable solid fractions (in most samples). At the same time, the intensity of the yellow color increased significantly, and a non-evaporating dissolved residue was detected. Figure 5A shows the dependence of the mass of the residual solid fraction on the destruction time. The mass is given as a percentage of the initial mass of polypropylene granules. Figure 5B corresponds to the approximation of the obtained dependence with the first-order kinetic model of polymer decomposition; m and m_0_ are the current and initial masses of the solid fraction.

Two stages with different decomposition rate constants *k*_1_ and *k*_2_ can be distinguished. The first stage in the interval of 0–3 h correlates with the time of visual disappearance of solid polymer granules in the mixture (Figure 2). According to the literature discussed above, the decomposition rate strongly depends on the structure and characteristics of the polymer (stereoregularity, degree of crystallinity, presence of side groups and branches (defects), aggregate state), as well as its ability to absorb oxygen. At the beginning of decomposition, the hydrophobic and lightweight PP granules float at the phase boundary of the oxygen-enriched water and phase of water vapor and O_2_. During oxidation, the polymer becomes more hydrophilic and the permeability of oxygen in PP may increase. It is interesting to note that the first stage of polymer decomposition in H_2_O/O_2_ mixture correlates with the stages of the acids formation (which may be dominantly influenced by hydroperoxides decomposition) (Table 1): $k_{total}$ = 17 ± 5 × 10^−5^ s^−1^ and $k^{H_{2}O}$_1_ = 17 ± 2 × 10^−5^ s^−1^ (Figure 5B). At 6 h and more, a homogeneous colorful acidic solution was obtained, thus the studied solid fraction corresponded only to hydrophilic oligomers with correspondingly slow kinetics of decomposition.

**Figure 6:**
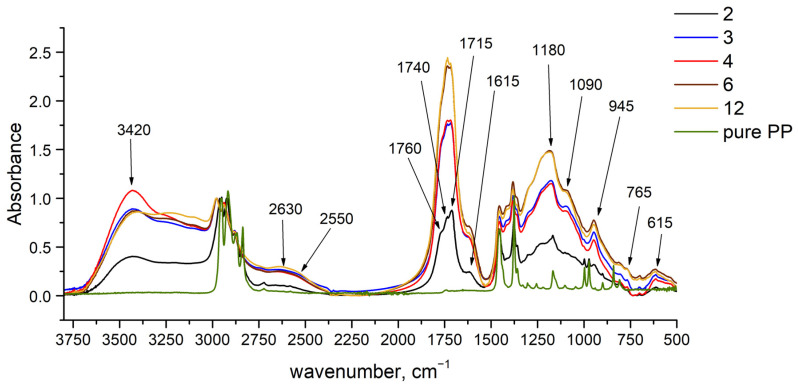
Typical FTIR spectra of samples obtained by TOD of PP in H_2_O/O_2_ mixture at different destruction times (in hours) and pure polypropylene.

FTIR spectra were also obtained for the solid fraction. Figure 6 shows the spectra of dried residues obtained by destruction for 2, 3, 4, 6 and 12 h. At longer destruction times, the IR spectra remain virtually unchanged. The figure shows the spectrum of pure polypropylene, and also the bands that appear in the destruction products but are absent in the pure polymer are indicated. All data are normalized to the band at 2950 cm^−1^, corresponding to C-H stretching vibration in CH_3_. According to Ref. [79] we assume that the band at 3420 cm^−1^ corresponds to valence vibrations of hydroperoxides. Bands in the region of 3500–3000 cm^−1^ could be attributed to vibrations of hydroxyl and hydroperoxide groups and also the N-H bond, but elemental analysis showed the absence of nitrogen in the dried residues (discussed in more detail below). It is clearly seen that between 2 and 4 h of destruction, the intensity of these bands increases sharply, reaching a maximum at 4 h of destruction, and then decreases slightly and does not change further. It can be assumed that the slight decrease in these bands is due to a decrease in the amount of hydroperoxides due to thermal decomposition [50]. The band at 765 cm^−1^ can also be attributed to vibrations of the hydroxyl group (tertiary ROH) [79]. The band that appeared at 615 cm^−1^ is ascribed to O–H vibrations in OH groups [80]. Bands in the region of 2650–2500 cm^−1^ can be interpreted as a manifestation of the hydrogen bonds between –COOH groups [81]. The band of 1780–1710 cm^−1^ is related to vibrations of carbonyl groups. According to the literature, carbonyl bands can be divided according to the structure of the molecule in which the group is present, with esters at ~ 1745 cm^−1^, ketones at 1725–1718 cm^−1^, and carboxylic acids at 1760–1705 cm^−1^ [79]. The noticeably growing bands at 1180 cm^−1^ and 1090 cm^−1^ are related to vibrations of the C-(CO)-C bond and C-O bond, respectively [82]. The band in the region of 1615 cm^−1^ corresponds to vibrations of the C=C bond [82,83]. Also, the characteristic bands corresponding to this bond may include the band at 3100 cm^−1^ (olefinic =C-H stretching) and 945 cm^−1^ (out-of-plane bending of olefinic C-H) [79]. The presence of unsaturated bonds may be one of the reasons for the observed color of the solutions in the case of their conjugation. The presence of double bonds is also confirmed by the qualitative reactions described below. On the other hand, no bands corresponding to aromatic compounds were found in the spectrum. Reliable determination of the structures responsible for the coloration of solutions is a separate complex task, beyond the scope of this study. However, it can be noted that some researchers involved in the oxidation of polyolefins suggest that the products of polyolefin oxidation that give coloration are carbon quantum dots [84]. Finally, it is important to note that in samples subjected to destruction for 3 h or more, the band at 2835 cm^−1^, corresponding to the valent asymmetric C-H vibrations of the methylene group, disappears, which indicates a gradual destruction of the main chain in the polypropylene structure. Similarly, at destruction times of over 3 h, the bands at 2915 cm^−1^ and 2950 cm^−1^ shift to the short-wave region at 2940 cm^−1^ and 2980 cm^−1^, respectively. This shift is observed in particular in the CH_3_ and CH_2_ groups located near the double C=C bond [85]. According to the IR data, it is clearly visible how the polymer is being destroyed over time. The number of oxygen-containing groups increases, and the bands corresponding to the vibrations of pure PP decrease.

For a more complete characterization, dried solid residues from polymer destruction for 3, 12 and 72 h were analyzed using a combination of other methods. According to the GPC data (Figure S3, Table S1), during the destruction from 12 to 72 h, the spread of the molecular weight M_w_ of the oligomeric fraction is from ~75,000 Da to ~3500 Da (it is also very likely that there is a fraction less than 1000 Da, but its separation with the column used is impossible). During the 3-h destruction, the maximum molecular weight is 60,000 Da. The peaks are quite smeared and it is impossible to determine the main fraction. No significant trends were revealed in the destruction process. The oligomeric fraction of the lowest molecular weight of 3500 Da corresponds to a model chain of pure polypropylene with a length of ~80 propylene units. The same length is obtained if we take into account that in every second unit, instead of a methyl group, there is a carbonyl group. The elemental analysis data (Table S4) show that the samples contain a large amount of oxygen (36% by weight), i.e., there is one oxygen atom per every two carbon atoms. The amount of hydrogen was 7% by weight, which in terms of conversion means one and a half hydrogen atoms per one carbon atom. Such a small amount may indicate that the samples contain a significant number of C=C bonds, identified by IR spectroscopy. The presence of carbon–carbon double bonds was also confirmed by the qualitative reactions. Firstly, it was shown that the addition of the water-soluble solid fraction of the PP decomposition product into a 3 vol. % aqueous solution of anhydrous FeCl_3_ leads to the formation of a red-colored suspension (Figure S6) due to the possible presence of enolic forms in oligomers. The color loss of the decomposition samples with the addition of bromine water was also observed (Video S1). The color loss may be induced not only by C=C bonds but also by the above-mentioned aldehyde groups. Their presence makes various re-condensation reactions probable. It is also important to note that elemental analysis did not reveal any nitrogen in the composition of the destruction products, so the color of the solutions should not be explained by the presence of any nitrogen-containing groups that could appear due to the presence of various functional additives in the polymer (antistatic, antioxidant, flame-retardant, etc.).

**Figure 7:**
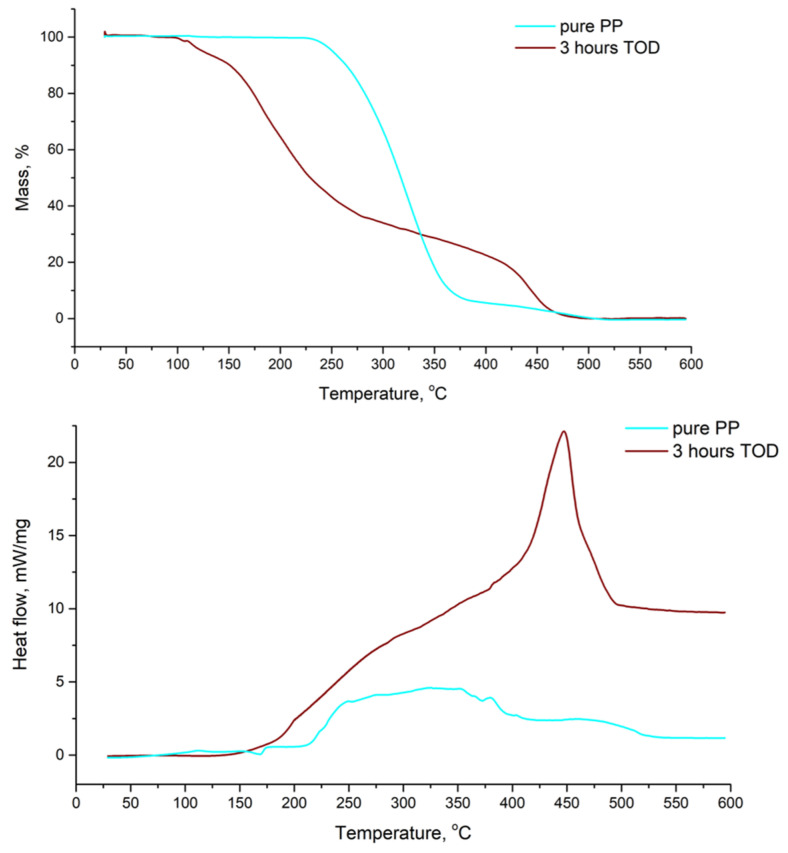
TGA (up) and DSC (down) data for sample obtained by TOD of PP in H_2_O/O_2_ mixture for 3 h and pure PP.

Figure 7 shows the TGA and DSC data for the sample subjected to 3-h destruction. The sample mass begins to decrease upon reaching 100 °C, which is due to the evaporation of residual water (the sample was initially dried at 40 °C in a vacuum, so some water could remain in it). Then the sample monotonously loses mass, which is explained by the gradual decomposition of residual oligomers. At about 250 °C, the mass loss rate slows down, but at this temperature no noticeable DSC peaks are observed. When the temperature reaches 250 °C, the destruction of pure PP begins, while the TOD products by this point have already lost more than 50% of their mass. A decrease in the temperature of initial mass loss indicates a decrease in the molecular weight of the solid polymer residue obtained during the TOD. Upon reaching 440 °C, acceleration of mass loss is observed. According to DSC data, a significant exothermic peak is observed at this temperature. In pure polypropylene, there is no such pronounced peak; however, weight loss in this area is also observed. It can be assumed that this difficult-to-decompose fraction is also present in the original polypropylene, and in the sample subjected to destruction, the specific amount of this fraction is just significantly higher, which makes its decomposition noticeable on the DSC and TGA curves. The presence of several mass loss areas for the 3-h sample indicates differences in the composition of its solid fraction.

### 3.6. The Composition of the Gas Phase Products Obtained in the Water-Assisted TOD Process of PP and Carbon Balance

For the samples with a decomposition time of 72 h, the gas phase of the products was For the samples with a decomposition time of 72 h, the gas phase of the products was also analyzed. The mass of the gas fraction for a series of such samples was 330 ± 30 mg. At the same time, the mass of oxygen initially pumped into the autoclave was 390 ± 10 mg. This reduction is expected, since a significant portion of the oxygen atoms after the process remains in the structure of the non-volatile fraction of the products. It was shown that the main component of the gas phase is unreacted oxygen (75%). The second component produced is CO_2_ (23%). Trace amounts of CO (0.9%) and N_2_ (0.5%) were also detected (a typical chromatogram is shown in Figure S9). The observed nitrogen can be attributed to residual air, which is apparently trapped in the autoclave, even after purging with oxygen. It is also important to note that the exhaust gas produced during the process contains only trace amounts of CO, which is comparable to the CO output in the process of high-temperature pyrolysis requiring much higher energy costs [86]. It should also be emphasized that, obviously, only a small part of the oxygen in the autoclave takes part in the oxidation of the polymer. However, as our experiments have shown, at a lower oxygen content (lower partial pressure in the autoclave), the process is slower (polymer granules disappear over longer periods of time) and results in a higher yield of residual oligomeric fraction. Based on the data of this work, it can be assumed that a higher oxygen partial pressure can accelerate the decomposition process in two ways: firstly, by ensuring its higher concentration upon dissolution in water (to increase the oxidative activity of such a liquid environment), and secondly, by promoting a higher diffusion flow of oxygen under pressure directly into the solid polymer located on the surface of the water. Finally, we emphasize that the remaining oxygen can be separated from CO_2_ and CO [87] and used in subsequent iterations of polypropylene processing, which will significantly reduce the cost of the process, since according to calculations, the portion of oxygen available for recycling is still 65% of the initial amount.

Using the obtained data on the composition of the gas phase, it is also possible to calculate the pressure achieved in the autoclave during the decomposition process. All calculations presented were performed using the NIST Chemistry WebBook program (National Institute of Standards and Technology, Gaithersburg, MD, USA). We consider the amount of water in the process to be constant (possible deviations in the real process will not have a noticeable effect on the final values). When reaching 150 °C, the water vapor in an autoclave will create a partial pressure of 4.7 bar. The pressure of initial pure oxygen will in turn increase by 9 bar. The final pressure in the autoclave will be 34 bar. If we now take into account that some of the oxygen will be consumed for the formation of acids and oligomers, and CO_2_ will also be produced, then the total pressure in the system will be about 33 bar (23 bar from the remaining pure oxygen, 4.5 bar from the formed CO_2_, and 4.7 bar from water vapor). Thus, a change in the composition of the gas products does not lead to a noticeable change in the pressure in the autoclave.

Based on the data from the analysis of the gas, liquid, and oligomeric fractions of the TOD products, a carbon balance was compiled for the samples that had been treated for 72 h. According to the calculations, 40% of the carbon atoms from the number initially contained in polypropylene end up in the composition of acids. The main contribution is made by the produced acetic acid, which contains about 35% of the carbon atoms. The generated CO_2_ accounts for 58% of the carbon atoms. CO accounts for less than 1%. The remaining carbon is thus contained in the oligomeric fraction. The number nearly coincides with the mass and elemental analysis data for the oligomeric fraction, taking into account that the proportion of carbon in the oligomeric fraction for 72 h of decomposition will not be greater than that of 3 h (which was studied by elemental analysis).

### 3.7. The Role of Water in the Studied TOD

**Figure 8:**
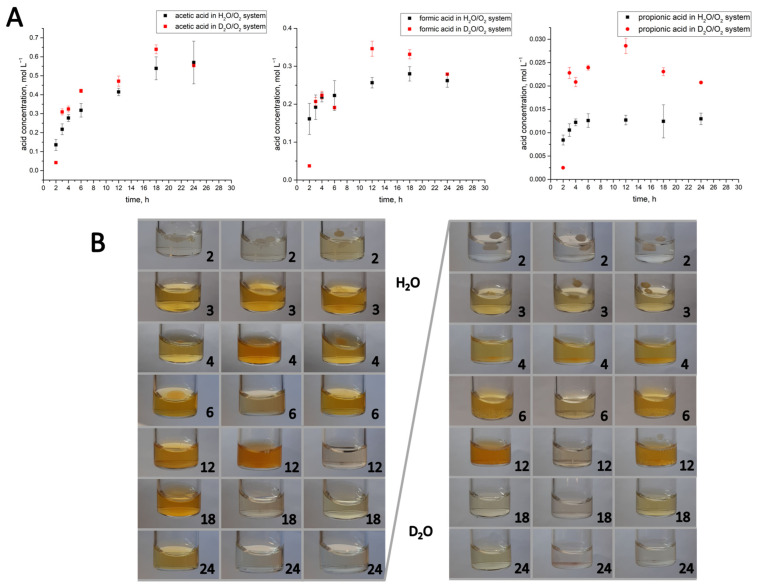
(**A**) Comparison of the kinetics of carboxylic acids formation in TOD in H_2_O/O_2_ and D_2_O/O_2_ systems. (**B**) Photographs correspond to samples obtained by TOD of PP in H_2_O/O_2_ and D_2_O/O_2_ mixtures at 2–24 h.

It is also interesting to discuss the role of the hot pressurized oxygen-enriched water in the studied TOD process, in particular, whether it acts only as a physical solvent or can participate in the formation of the active species. For this purpose, we studied the kinetics of acids formation in both H_2_O and D_2_O systems (Figure 8A).

In wet oxidation systems, intermediate active radical species can be formed in the reaction between oxygen and water (HOO^•^, HO^•^) and oxidize polymer [44,88]; their formation depends on the pressure and temperature conditions, the catalyst, dissolved oxygen concentration, autoclave geometry, the composition of the autoclave walls, the solution pH, and the “nature” of the organic compounds (as well as intermediates formed)/types of chemical reactions that occur [44]. The chosen conditions (20 bar, 150 °C) of our process are similar to those of wet oxidation systems [44]. Moreover, despite the use of a PTFE vessel, pressurized oxygen and water vapor in our system still contact with steel walls of the autoclave. According to Ref. [89], hydroxyl radicals (HO^•^) and hydrogen peroxide (H_2_O_2_) intermediates were detected directly by means of electron spin resonance spectroscopy (ESR) during non-catalytic wet air oxidation of cellulose (260–320 °C with added air-O_2_ at 25–40 bar). In the catalytic wet air oxidation (WAO) of chloroquine phosphate the superoxide radical (O_2_^−•^) and hydroxyl radicals (HO^•^) were detected through ESR analysis, at oxygen pressure of 15 bar and temperature of 230 °C [90]. The study of temperature-dependent free radical reactions using nitroxyl radicals as redox probes suggested that heating an aqueous solution containing oxygen can generate O_2_^−•^ [91].

The Bruskov group studied the formation of hydrogen peroxide in hot oxygen-enriched water [92,93] and speculated on the reaction pathway through singlet oxygen as the starting source of active radical species formation: O_2_ → ^1^O_2_ → O_2_^−•^ → HO_2_^•^ → H_2_O_2_ → HO^•^. They observed an increase in H_2_O_2_ concentration while substituting H_2_O with D_2_O, which could increase the lifetime of the singlet oxygen by an order of magnitude. In our experiments, the substitution of H_2_O with D_2_O resulted in a sharp increase in the rate of acid formation from 2 to 3 h, which may indicate a more active formation (or increased lifetime) of radical species oxidizing polymer and intermediates in the heavy water system (Figure 8A).

A comparison of the appearance of decomposition products obtained from the TOD of PP in H_2_O/O_2_ and D_2_O/O_2_ mixtures is presented in Figure 8B. At the first stage of the process (corresponding mainly to the oxidation of the initial solid PP for 0–2 h), the decomposition of solid PP granules in a D_2_O/O_2_ mixture slows down slightly: it can be seen that after 2 h of TOD in heavy water enriched with O_2_, the PP granules show less decomposition. This observation correlates with the observed low concentrations of acids at *t* = 2 h in the case of heavy water in Figure 8A and may be explained by ~20% slower diffusion of O_2_ molecules in more viscous D_2_O [94]. Then, from 2 to 3 h, solid PP residues are still obtained in the D_2_O/O_2_ system, while the heavy water mixture is less yellow, which correlates with higher concentrations of colorless/light yellow carboxylic acids in the D_2_O/O_2_ system (Figure 8A). This may be due to the more intense decomposition of intermediates into carboxylic acids in the system with heavy water. After more than 4 h, mixtures gradually lose yellow tint during decomposition. Nearly colorless products are obtained after 18 h in the D_2_O/O_2_ system, while a yellow color provided by the presence of oxidized oligomers in the solution is observed in some samples even after 24 h in the H_2_O/O_2_ system (Figure 8B). This directly observable and effective acceleration of the decomposition process at the final stage in heavy water solution is also consistent with an increase in acid concentrations after 3 h (Figure 8A).

## 4. Conclusions

The paper considers in detail the kinetics of carboxylic acids formation in the process of “green”, water-assisted thermal oxidative destruction of polypropylene in the presence of pressurized oxygen. It is shown that the main products of decomposition both at short times (2–20 h) and at long times (24–72 h) are acetic and formic acids, and the concentration of acetic acid relative to formic acid is constantly increasing. Therefore, the selectivity of the conversion improves with time. The correspondence of the kinetics of carboxylic acids formation to the first-order model at 2–6 h of the TOD process may be due to the dominant role of the decomposition of hydroperoxides, which limits the rate of formation of low-molecular products. After 6 to 18 h, the rates of acids formation seem constant and do not depend on their concentration.

The first stage of the monocarboxylic acids formation correlates with the onset of PP destruction, when the solid polymer granules in the mixture disappear and the acidic solution becomes colorful with dissolved hydrophilic oligomeric residues in it. At this stage, the rate of the polymer decomposition correlates with that of acid formation; the first-order kinetic constants of both polymer destruction and acid formation are in good agreement: $k^{H_{2}O}$_1_ = 17 ± 2 × 10^−5^ s^−1^ and $k_{total}$ = 17 ± 5 × 10^−5^ s^−1^. After complete conversion of the solid polypropylene granules into water soluble oligomers, the TOD process in the homogeneous solution slows down. The study of the TOD process in heavy water revealed the active role of water in the destruction reaction pathways. Finally, the solid dissolved residue remaining even after 72 h of destruction (1 wt.% of the initial polymer weight) was studied and characterized in detail. It was shown that the solid residue includes many C=C double bonds, while the absence of aromatic compounds indicates the absence of the formation of toxic polycyclic aromatic hydrocarbons, which are usually formed during the destruction of polyolefins and indicates the prospects of the studied water-assisted TOD method as a “green” technique for converting polypropylene into carboxylic acids. In turn, the gaseous products contain mainly unreacted oxygen and CO_2_ with only trace amounts of CO. Carbon balance calculations showed that about 40% of the initial polypropylene is processed into acids. It is likely that the choice of a suitable catalyst can significantly shift the reaction balance towards a higher yield of acids, which will be one of the important directions for the further development of this work. Despite the fact that this method of obtaining acids is obviously less economical than existing industrial technologies of their direct synthesis, this approach allows a fairly narrow distribution of acids, with a predominant content of acetic acid, which is in demand in the chemical industry, to be obtained from polypropylene waste and not a typical low-grade fuel with a wide distribution of fractions.

Substitution of H_2_O with D_2_O in the TOD process leads to the slowing down of the first stage of the process (corresponding mainly to the oxidation of initial solid PP at 0–2 h), which may be explained by the ~20% lower diffusion of O_2_ molecules in more viscous D_2_O. Then, at TOD times of 2–3 h, a sharp increase in the rate of the carboxylic acid formation is observed, which can indirectly confirm the participation of heavy water in the active species formation in the studied system. The data presented in this paper contribute to the development of eco-friendly and safe chemical methods for polypropylene processing and the transfer of these approaches to real technologies.

## Figures and Tables

**Table 1:** The results of the fitting of the obtained acids concentration on time dependencies with different kinetic models.

Product	First-Order Kinetics of the TOD Process at t = 2–6 h and the Values of $k_{acid}$
acetic + formic + propionic + succinic acids	$k_{total}$ = 1.7 ± 0.5 × 10^−4^ s^−1^; R^2^ = 0.97
acetic acid	$k_{acetic}$ = 1.5 ± 0.3 × 10^−4^ s^−1^; R^2^ = 0.99
formic acid	$k_{formic}$ = 2.3 ± 1.3 × 10^−4^ s^−1^; R^2^ = 0.89

## Data Availability

The data presented in this study or Supplementary Materials are available upon request.

## Supplementary Materials

The following supporting information can be downloaded at: https://www.mdpi.com/article/10.3390/polym17192696/s1, Figure S1. Photographs of series of samples obtained by TOD of PP at different destruction times (in hours), Figure S2. Visible spectra of the samples obtained at different thermal oxidation times in H_2_O/O_2_ mixtures. The graphs show the typical absorbance peaks in UV range for all samples. UV spectra were measured for diluted 0.6 vol.% aqueous solutions of the samples, Figure S3. GPC data for the samples obtained by thermal oxidative decomposition of polypropylene at different destruction times (in hours), Figure S4. Chromatogram of the solution obtained by TOD for 72 h, Figure S5. Electropherograms of the solution obtained by TOD for 18 h, Figure S6. The photographs of the aqueous mixtures of the oligomeric solid residues obtained after 18 h of water-assisted TOD process and solution FeCl_3_, Figure S7. The photographs of the reaction products of liquid fractions obtained in the TOD process after 6 h (1) and after 72 h (2) with Cu(OH)_2_ in alkaline solution, Figure S8. The photographs of the reaction products of liquid fractions obtained in the TOD process after 6 h (tube 2) and of the aqueous solution of the oligomeric solid residues obtained after 18 h of the water-assisted TOD process (tube 3) with KI solution (reaction time 24 h), Figure S9. Typical gas phase chromatogram of decomposition products, Figure S10. Photographs of samples obtained by TOD of PP at 150 °C for 72 h at different oxygen pressures, Figure S11. Photo of a PTFE cylinder. Table S1. Molecular weight distribution of solid residues calculated based on GPC data, Table S2. Acids identified by GC–MS and corresponding release times, Table S3. Metrological characteristics of the capillary electrophoresis method for determining acetic, formic, succinic, and propionic acids; Table S4. Mass and molar fraction of elements in the oligomeric residue obtained during the destruction of PP for 3 h according to elemental analysis data; (Video S1, mp4). Video of the interaction of liquid PP decomposition products with bromine 0.18 M solution.

## Abbreviations

The following abbreviations are used in this manuscript:

DSC	Differential scanning calorimetry
FTIR	Fourier IR spectroscopy
GC-MS	Gas chromatography–mass-spectrometry
GPC	Gel permeation chromatography
NMR	Nuclear magnetic resonance
OD	Oxidative decomposition
PE	Polyethylene
PP	Polypropylene
PTFE	Polytetrafluoroethylene
TGA	Thermogravimetric analysis
TOD	Thermal oxidative decomposition
UV	ultraviolet
