# Supplementary material for: Kinetics of Carboxylic Acids Formation During Polypropylene Thermooxidation in Water Saturated with Pressurized Oxygen
Source: Polymers (Basel). 2025 Oct 7;17(19):2696. doi: 10.3390/polym17192696 (PMC12526518; doi:10.3390/polym17192696)
Supplement: Supplementary file 1 [file polymers-17-02696-s001.zip › supplementary_PP_Pol_revised_2.pdf]

Kinetics of Carboxylic Acids Formation During Polypropylene Thermooxidation in Water Saturated with Pressurized Oxygen

Vadim V. Zefirov [1,*], Polina S. Kazaryan [1], Andrey I. Stakhanov [1], Svetlana V. Stakhanova [2], Mikhail M. Ilyin [1], Ivan A. Godovikov [1], Elizaveta V. Shmakova [2], Andrey G. Terentyev [2], Alexander V. Dudkin [2], Elena P. Kharitonova [3], Marat O. Gallyamov [1,3] and Alexei R. Khokhlov [1,3]

Supplementary materials

Figure S1 shows photographs of a series of samples obtained during polypropylene destruction at different exposure times: from 2 to 72 hours. It is clearly seen that within one series (one destruction time) the samples somewhat differ in color intensity. This is observed even at 72 hours of destruction. At the same time, the masses of dried residues at such times differ only slightly, as do the concentrations of acids in solutions. Therefore, it can be assumed that the total amount of dissolved substances providing color in the samples is small.

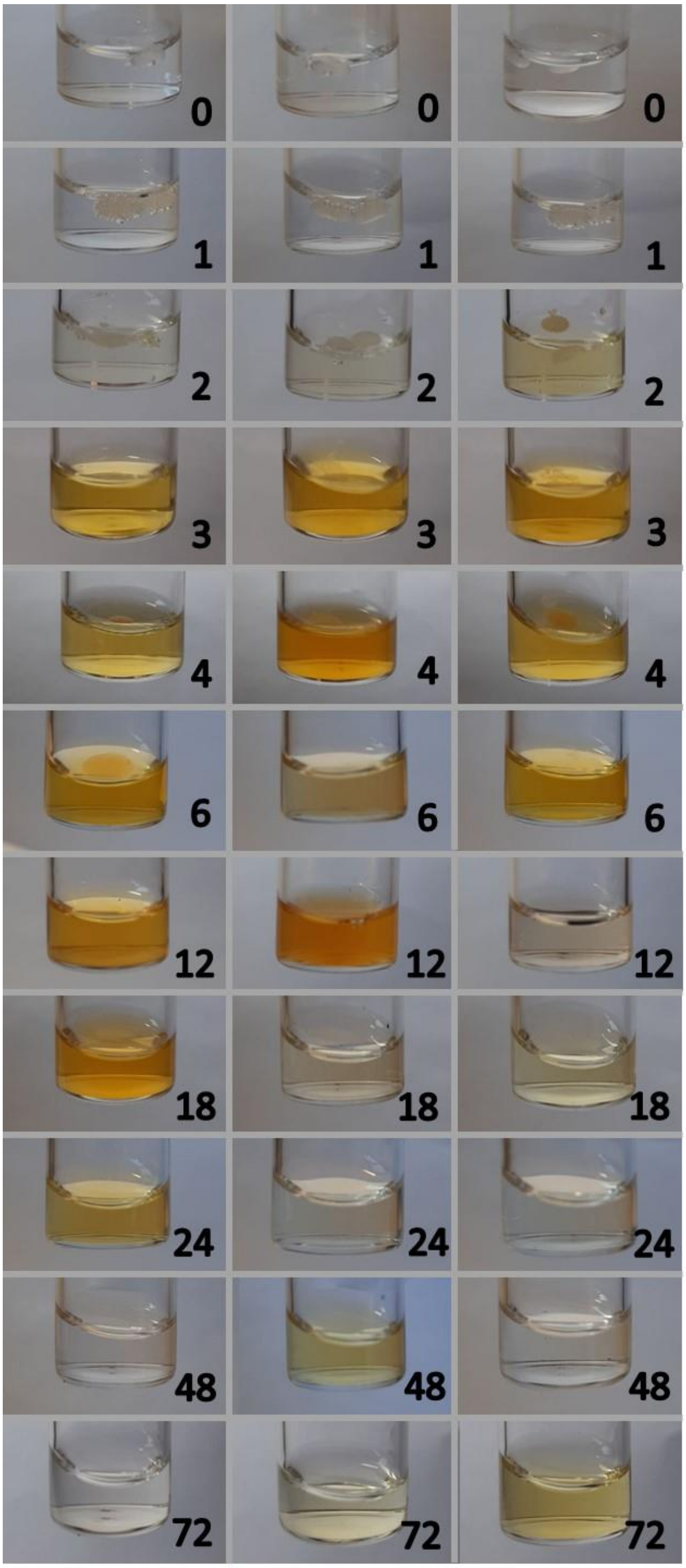

Figure S1. Photographs of series of samples obtained by TOD of PP at different destruction times (in hours).

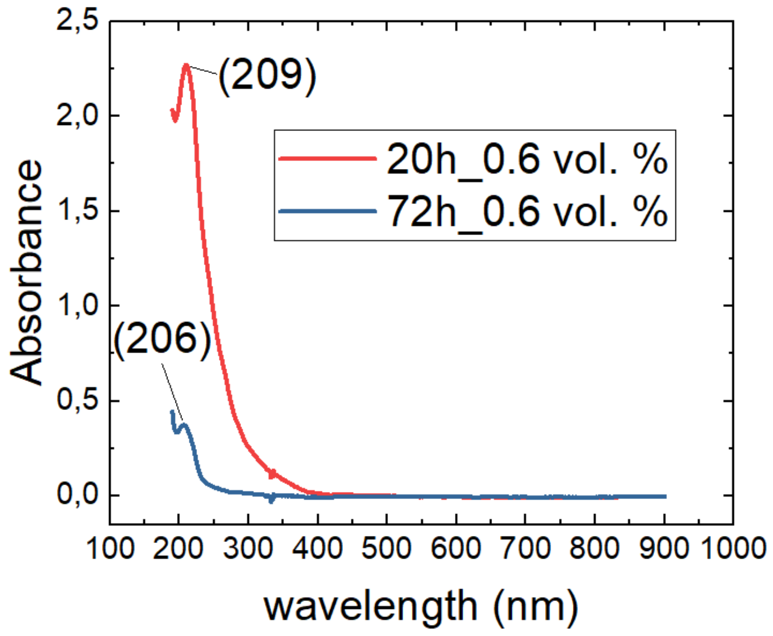

Figure S2. Visible spectra of the samples obtained at different thermal oxidation times in H_2O/O_2 mixtures. The graphs show the typical for all samples absorbance peaks in UV range. UV spectra were measured for diluted 0.6 vol.% aqueous solutions of the samples.

Figure S3 shows the GPC graphical data for the non-evaporative residual fraction of the samples obtained by degradation for 3, 12 and 72 hours.

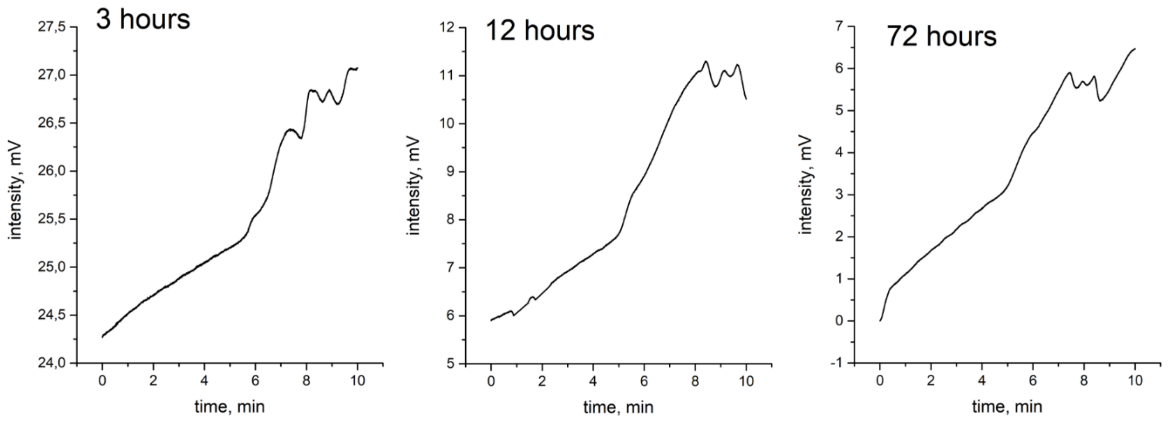

Figure S3. GPC data for samples obtained by thermal oxidative decomposition of polypropylene at different destruction times (in hours).

Table S1. Molecular weight distribution of solid residues calculated based on GPC data.

Destruction time, hours	Retention time, min	Mn, Da	Mw, Da	distribution coefficient D
3	6.313	58746	60203	1.025
	7.315	23324	25546	1.095
	8.150	10267	10663	1.039
	8.895	5943	6081	1.023
	9.727	3385	3500	1.034
12	6.127	75176	78881	1.049
	8.102	19832	24292	1.225
	8.405	8356	8571	1.026
	9.112	5181	5287	1.020
	9.640	3278	3365	1.027
72	6.182	72259	76183	1.054
	7.352	25930	29049	1.120
	7.907	13040	13247	1.016
	8.373	9585	9702	1.012
	9.720	3475	3646	1.049

The practical application of the GC-MS method in the case of aqueous solutions of carboxylic acids is complicated by the fact that due to the high activity of hydroxyl groups, the substances being studied adsorb in the injector and chromatographic column, which results in the transformation of the substances, their loss and distortion of the results of quantitative determination. To avoid this, during identification, the analyzed components were first transferred to the organic phase by extraction, and then, if necessary, subjected to

derivatization, *i.e.*, the conversion of polar hydroxyl groups into non-polar ones without disrupting the main structure of the substance. Ethyl acetate was used to extract acids from the aqueous solution. The sample volumes ranged from 500 µl to 100 µl, depending on the amount available. The extraction solvent was added in a 1:1 ratio. After extraction, drying was performed with anhydrous calcined $MgSO_4$. N,O-bis(trimethylsilyl)trifluoroacetamide (BSTFA) was used as the silylating agent. To obtain silyl derivatives, 100 µl samples were taken from the dried solution, to which BSTFA was added in a volume ratio of 1:1. To ensure complete reaction, the mixture in closed vials was kept at 70°C for 30 minutes. The silylation was chosen as a method for derivatization of compounds containing a hydroxyl group as far as the advantages of the resulting silyl derivatives are that they are more volatile, provide good separation on the chromatogram, intense peaks in the mass spectrum, and ensure high sensitivity during determination. Chromatography was carried out under the following conditions: a 12–25 m long quartz capillary column coated with a methylsiloxane phase was used, the evaporator temperature was 280 °C, the detector temperature was 290 °C, and the column temperature was changed from 200 to 280 °C at a rate of 10 deg/min. The carrier gas was helium. The mass spectra were interpreted using NIST databases.

Below is the chromatogram of solutions obtained during the destruction of PP and table of identified components for a sample subjected to destruction for 72 hours.

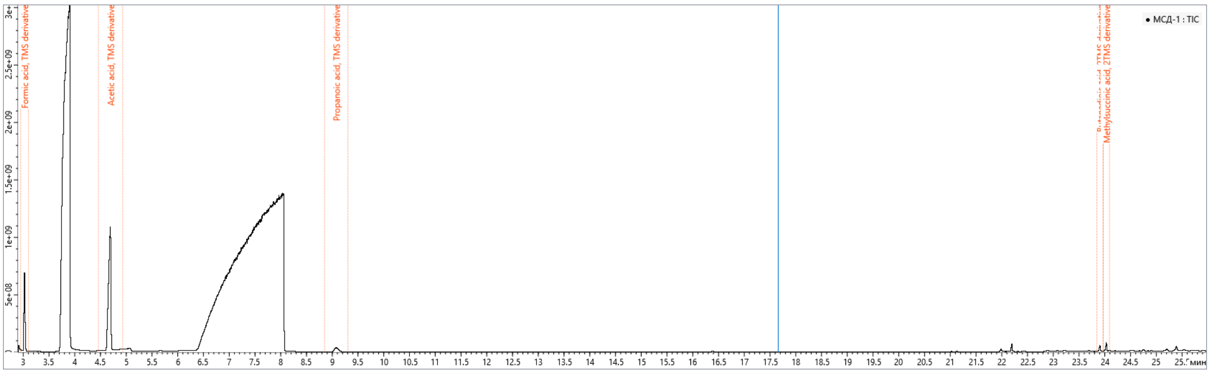

Figure S4. Chromatogram of the solution obtained by TOD for 72 hours.

Table S2. Acids identified by GC-MS and corresponding release times.

Acid	Release time, min
Formic acid	2.995
Acetic acid	4.580
Propionic acid	8.835
Succinic acid	23.865
Methylsuccinic acid	23.990

Capillary electrophoresis was used to quantify the content of carboxylic acids. A capillary electrophoresis system CAPEL-105 M (Lumex, Russia) equipped with a spectrophotometric detector and a quartz capillary tube (internal diameter 75 µm, effective length 50 cm, total length 60 cm) and Elforun® software (Lumex, Russia) was used. The assay was carried out under the following conditions: the capillary temperature was thermostatically controlled at 25 °C; the voltage applied to the capillary tube was −20 kV; hydrodynamic injection of the sample was performed at 30 mbar for 12 s. The background electrolyte composition was 10 mmol/l benzoic acid with the addition of 0.5 mmol/l cetyltrimethylammonium hydroxide and sodium hydroxide with pH 8.6. Indirect UV detection was used at 254 nm wavelength. Between the series of runs the capillary was rinsed for 5 min with water, a 1 M solution of HCl for 5 min, water for 5 min, a 1 M solution of NaOH for 5 min, and water for 5 min again. Probes of the liquid fractions obtained in the TOD process were centrifuged at 12,000 rpm for 5 min and diluted 100-fold with Milli-Q purified water before analysis. To obtain quantitative results, the external calibration method was used.

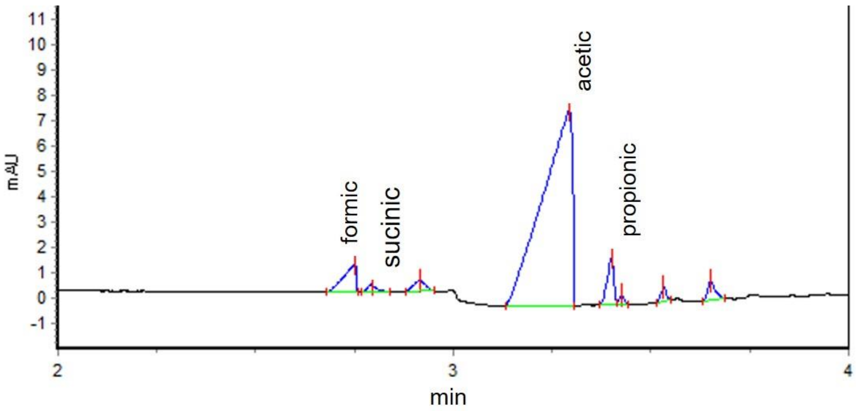

Figure S5. Electropherograms of the solution obtained by TOD for 18 hours.

Table S3. Metrological characteristics of the capillary electrophoresis method for determining acetic, formic, succinic and propionic acids (n = 5, P = 0,95)

Acid	Calibration equation	Coefficient of determination, R^2	Detection limit, µg/ml	Limit of quantification, µg/ml
Acetic	$C = 0,4314s$	0,9973	0,016	0,053
Formic	$C = 0,5351s$	0,9947	0,011	0,038
Succinic	$C = 0,5887s$	0,9946	0,005	0,018
Propionic	$C = 0,2047s$	0,9974	0,015	0,030

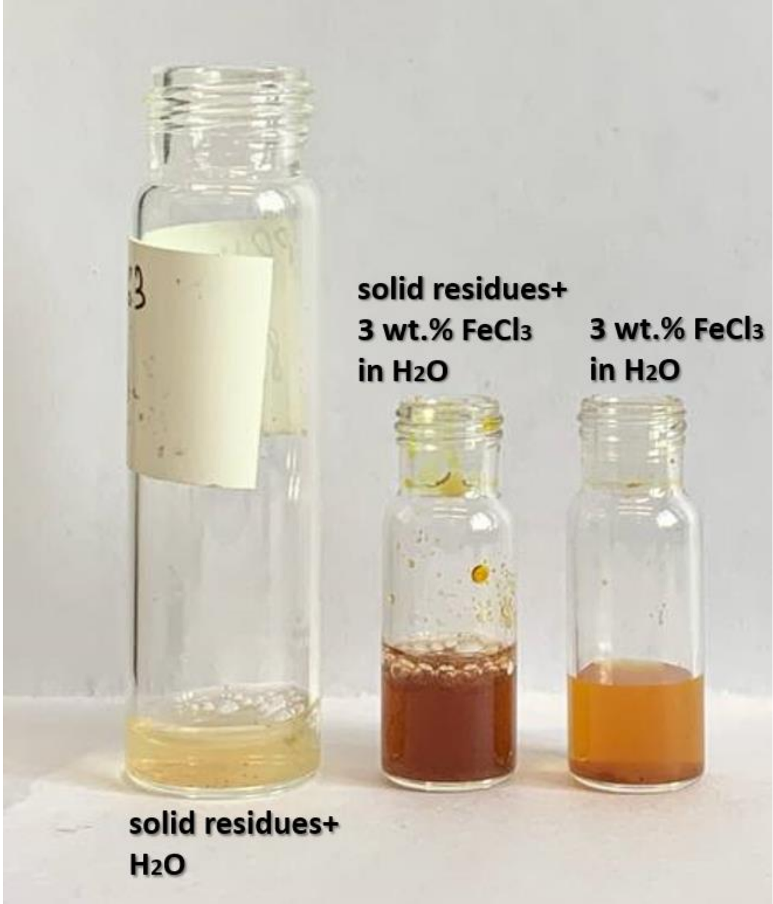

Figure S6. The photographs of the aqueous mixtures of the oligomeric solid residues obtained after 18h of water-assisted TOD process and solution $FeCl_3$.

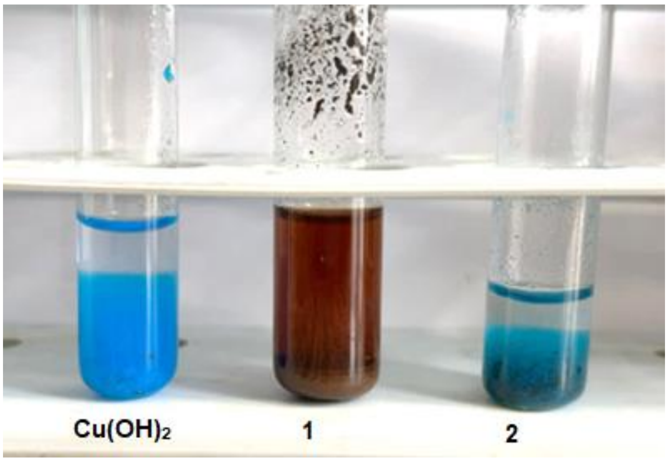

Figure S7. The photographs of the reaction products of liquid fractions obtained in the TOD process after 6 h (1) and after 72 h (2) with $Cu(OH)_2$ in alkaline solution.

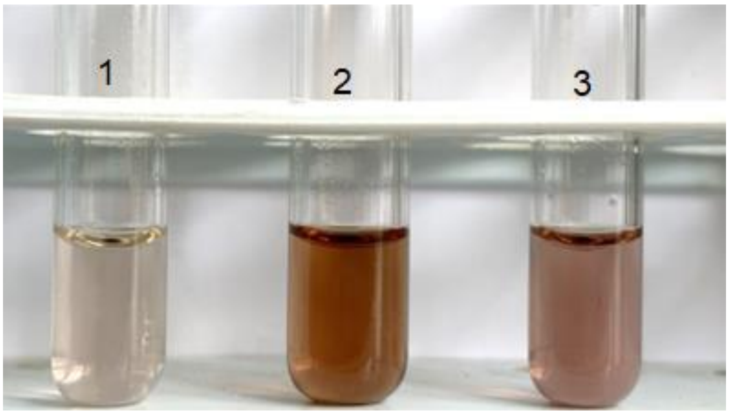

Figure S8. The photographs of the reaction products of liquid fractions obtained in the TOD process after 6 h (tube 2) and of the aqueous solution of the oligomeric solid residues obtained after 18h of water-assisted TOD process (tube 3) with KI solution (reaction time 24 h). Test tube 1 shows the result of keeping the same solution of KI in contact with atmospheric oxygen. Starch was used as an indicator.

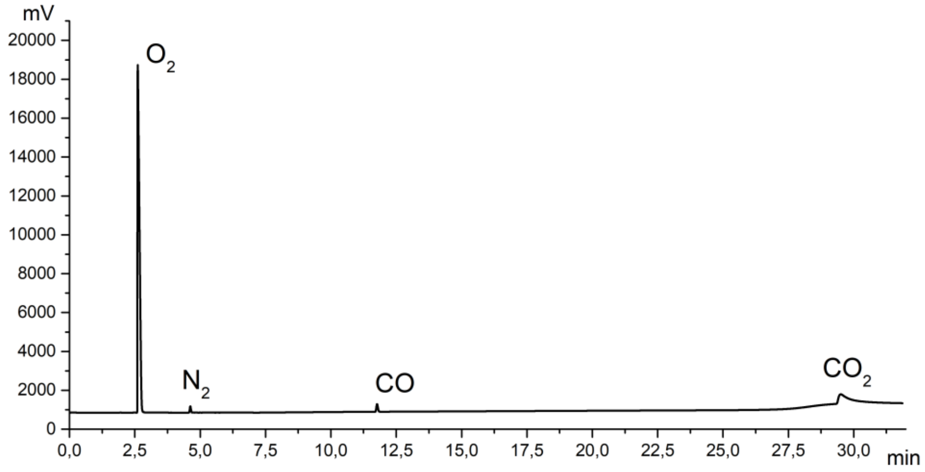

Figure S9. Typical gas phase chromatogram of decomposition products.

To select the synthesis parameters, experiments were carried out at different oxygen pressures. Figure S10 shows images of samples obtained by decomposition at a pressure of 10, 15 and 20 bar at a temperature of 150 °C for 72 hours. The process temperature was chosen based on our previous work, which showed that polymer conversion decreases as the temperature decreases. As the experiments described in the work showed, the color of the samples correlates with the amount of residual oligomeric fraction: the higher the color intensity, the more oligomeric fraction remains in the sample. Thus, it is evident that a pressure of 20 bar provides the minimum residual amount of oligomeric fraction. At the same time, with a successive increase in pressure to 60 bar, no changes in the color of the resulting solutions were detected.

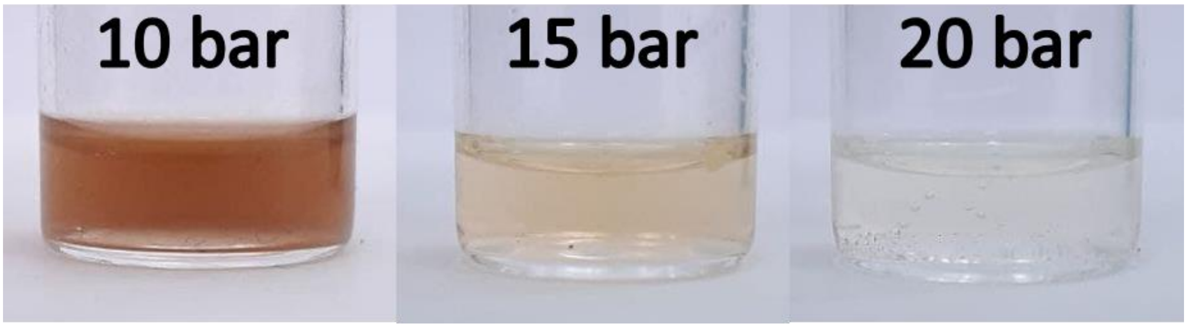

Figure S10. Photographs of samples obtained by TOD of PP at 150 °C for 72 hours at different oxygen pressure.

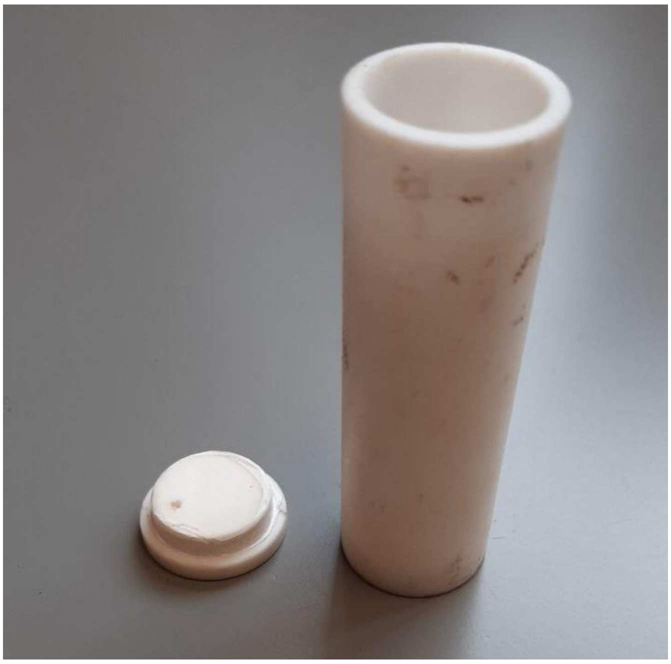

Figure S11. Photo of a PTFE cylinder

Table S4. Mass and molar fraction of elements in the oligomeric residue obtained during the destruction of PP for 3 hours according to elemental analysis data.

element	mass fraction	molar fraction
C	0.571 ± 0.001	0.333 ± 0.004
H	0.073 ± 0.004	0.51 ± 0.03
O	0.365 ± 0.005	0.156 ± 0.003
